# Mesoscale Characterization of Supramolecular Transient Networks Using SAXS and Rheology

**DOI:** 10.3390/ijms15011096

**Published:** 2014-01-16

**Authors:** A. C. H. Pape, Maartje M. C. Bastings, Roxanne E. Kieltyka, Hans M. Wyss, Ilja K. Voets, E. W. Meijer, Patricia Y. W. Dankers

**Affiliations:** 1 Institute for Complex Molecular Systems, Eindhoven University of Technology, PO Box 513, 5600 MB, Eindhoven, The Netherlands; E-Mails: a.c.h.pape@tue.nl (A.C.H.P.); maartje.bastings@gmail.com (M.M.C.B.); r.e.kieltyka@chem.leidenuniv.nl (R.E.K.); h.m.wyss@tue.nl (H.M.W.); i.voets@tue.nl (I.K.V.); e.w.meijer@tue.nl (E.W.M.); 2 Laboratory of Chemical Biology, Eindhoven University of Technology, PO Box 513, 5600 MB, Eindhoven, The Netherlands; 3 Department of Mechanical Engineering, Eindhoven University of Technology, PO Box 513, 5600 MB, Eindhoven, The Netherlands; 4 Laboratory of Macromolecular and Organic Chemistry, Eindhoven University of Technology, PO Box 513, 5600 MB, Eindhoven, The Netherlands.

**Keywords:** supramolecular polymers, rheology, hydrogels, small-angle X-ray scattering

## Abstract

Hydrogels and, in particular, supramolecular hydrogels show promising properties for application in regenerative medicine because of their ability to adapt to the natural environment these materials are brought into. However, only few studies focus on the structure-property relationships in supramolecular hydrogels. Here, we study in detail both the structure and the mechanical properties of such a network, composed of poly(ethylene glycol), end-functionalized with ureido-pyrimidinone fourfold hydrogen bonding units. This network is responsive to triggers such as concentration, temperature and pH. To obtain more insight into the sol-gel transition of the system, both rheology and small-angle X-ray scattering (SAXS) are used. We show that the sol-gel transitions based on these three triggers, as measured by rheology, coincide with the appearance of a structural feature in SAXS. We attribute this feature to the presence of hydrophobic domains where cross-links are formed. These results provide more insight into the mechanism of network formation in these materials, which can be exploited for tailoring their behavior for biomedical applications, where one of the triggers discussed might be used.

## 1. Introduction

Hydrogels are widely investigated for their use in biomedical applications in the field of regenerative medicine, for example as scaffolds for tissue engineering and as drug delivery vehicles. Hydrogels are potentially highly compatible with biological species such as proteins or cells [1,2]. To create materials tailored for biomedical applications, the relationship between biological function on the one hand and mechanical properties and morphology on the other hand, should be understood and optimized. Spatial and temporal control over the structure of the hydrogel is key to achieve this goal; for instance to position biological signals or to adapt the mechanical properties [3]. Obtaining control over the structure can be regulated via various triggers such as pH and temperature, which determine the applicability of the materials.

In supramolecular systems, such spatiotemporal control has been proposed to be offered by the inherent reversibility of the non-covalent interactions [4,5]. Supramolecular networks in water can be formed by any non-covalent interaction, for example transient networks can be formed by hydrophobically end-modified water-soluble polymers [6,7]. In addition, supramolecular polymers are held together by non-covalent, directional interactions such as hydrogen-bonding, π–π stacking or metal-coordination. A hydrophilic block ensures water solubility which causes the supramolecular polymer to self-assemble into nanostructures [8–12]. These different interactions can be used to switch the aggregation state of the separate molecules in the system using environmental triggers [13], which in turn can lead to dramatic changes of macroscopic properties, such as the transition from an elastic gel to a liquid-like solution [14,15]. Switching of the aggregation state allows for mixing and blending approaches under mild conditions, for example to tune gel properties and incorporate bioactive signals and drugs [2,16]. For compatibility with bioactive signals and drugs, diverse switching approaches and thus multi-responsive systems are desirable [17,18].

Despite the promising properties of hydrogels to be used as biomaterials, they generally lack the mechanical toughness and strength required for applications inside the body, where biotissues have moduli of 10^4^–10^7^ Pa [19,20]. Therefore, a large proportion of the research on hydrogels has focused on improving the mechanical properties and mimicking the mechanical behavior of biological tissue. However, research on supramolecular systems focused mainly on the assembly process at the molecular level into nano-objects [21]. While the control over the gel structure at different length scales is essential, little information is available on the microscale structures in these hydrogels. More research on the structure at these length scales, and relations between the different length scales, as well as on the mechanical properties, is therefore essential.

The combination of small-angle X-ray scattering (SAXS) and oscillatory rheology has proven to be a valuable tool in the study of aqueous polymer networks and their phase diagrams [22–24], but has not been extensively used in the study of supramolecular polymers. Both analysis techniques allow for investigation of the polymers in the gel state, and create an opportunity to match the mechanical properties of the gel with the underlying mesoscopic network morphology. In a study of flowerlike micelles, the combination of rheology and SAXS has enabled extracting information on the number of bridges between micelles, which could be directly related to the mechanical properties [22]. In another example, neutral and symmetric poly(styrene) poly(methoxy diethylene glycol acrylate) triblock copolymers showed stronger correlations between micelles upon increasing concentrations, and an increased volume fraction of correlated micelles, which could be related to the mechanical response of the material [23]. A combination of rheology and SAXS was also recently used to investigate complex coacervates, revealing a transition in the ordering of spherical domains from body-centered cubic to hexagonally packed cylinders with increasing concentration, which leads to a decrease in the storage modulus of the material [24]. These examples show that the combination of SAXS and rheology can lead to a better understanding of structure-property relationships, also in supramolecular systems.

**Figure 1.**
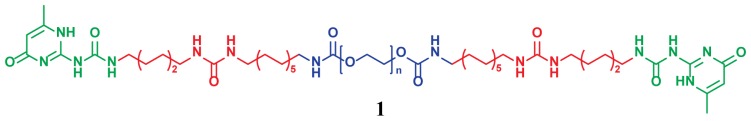
Chemical structure of the supramolecular UPy-PEG hydrogelator: poly(ethylene glycol) (blue, *M_n_* = 10 kDa, *n* ≈ 227 for **1a**; *M_n_* = 20 kDa, *n* ≈ 454 for **1b**) end-functionalized with ureido-pyrimidinone (UPy) groups (green), which are flanked by hydrophobic spacers containing urea groups (red).

Here, we aim to use these techniques for studying the structure-property relationship in transient networks in water during sol-gel transitions using different triggers such as pH and temperature. These networks are formed by supramolecular polymers developed in our group and consist of poly(ethylene glycol) (PEG), end-modified via a unique four-fold hydrogen bonding motif, the ureido-pyrimidinone (UPy) group (Figure 1) [25,26]. It is proposed that the UPy-groups dimerize and stack into fiber-like structures, facilitated by additional urea hydrogen bonding in a hydrophobic pocket formed by the alkyl spacer [16,18,25]. Concentration, temperature and pH can be used to introduce network formation in the system via physical cross-links, thereby switching from solutions to gels. We have recently demonstrated the benefits of such a physically cross-linked responsive system after minimally invasive application as a drug delivery vehicle in the heart [18] and under the renal capsule [25,26], using the pH responsiveness and the temperature, respectively. However, the exact mechanism of the assembly and hydrogelation in time has not been elucidated.

Better insight into the relation between structure and mechanical properties in these systems can potentially be exploited for tailoring the behavior of these systems to biomedical applications, where a precise control of mechanical behavior is required. Such understanding could benefit the development of optimized hydrogels that are both tough and strong, but also responsive to environmental triggers. Here we present a detailed characterization of the sol-gel transition of these UPy-PEGs at both the meso- and macroscopic scales, where we use SAXS to study changes in microstructure, and oscillatory rheology to follow the corresponding changes in viscoelastic properties.

## 2. Results and Discussion

### 2.1. Sol-Gel Transition Triggered by Concentration

Recently, we have shown that UPy-PEG molecules can form physically cross-linked networks [25,27]. The sol-gel transition was determined using the vial inversion test, where critical concentrations of 5 and 10 wt % for **1a** and **1b** were found, respectively, below which the gels could not support themselves in the vial. However, this test provides only limited information, as in typical vials (*Ø* = 1 cm) gels with a yield stress below 40 Pa will still flow [28], with the threshold to such yielding depending on the size of the vials and the mass of the sample used in the test. Initial rheology experiments for the samples indicated a storage modulus *G*′ that only weakly depends on frequency ω, with *G*′ considerably larger than the loss modulus *G*″ in the range of frequencies studied, as expected for elastic-like gels [25].

**Figure 2.**
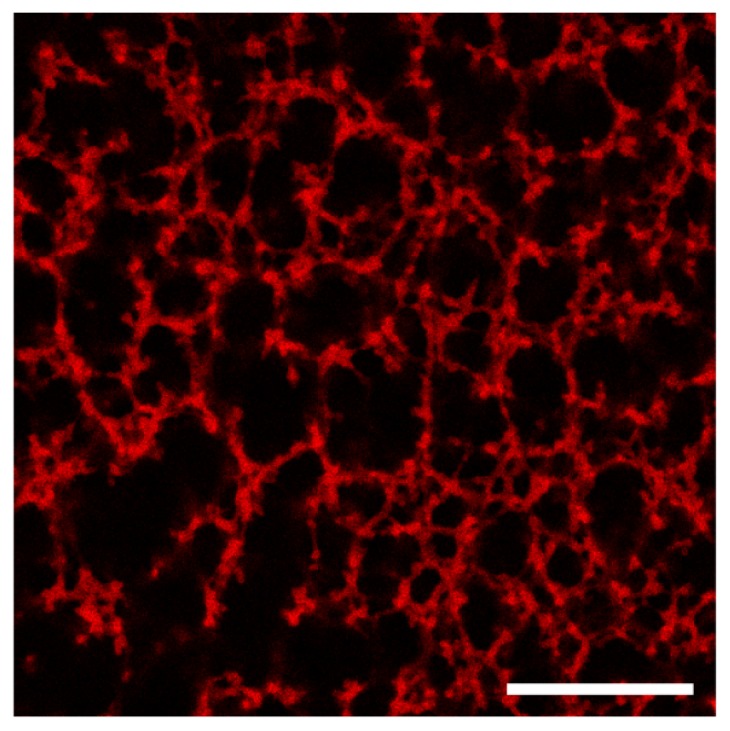
Confocal microscopy image of a viscoelastic solution of **1a** at a concentration of 1 wt % under neutral conditions. The scale bar corresponds to 50 μm.

Confocal microscopy using a solvatochromic dye (Nile red) was shown to be a valuable tool in the study of formation of a transient network [16]. Confocal microscopy imaging of our samples, with acquisition times around 1 second, indicates that even at concentrations as low as 1 wt % a network is formed in the material (Figure 2). However, the resolution of the microscope (on the order of a few hundreds of nanometers) is too low to distinguish smaller objects that might make up the network. Moreover, at higher concentrations, a homogeneous fluorescent signal is obtained, as the mesh of the network becomes too dense to be imaged using confocal imaging (Figure S1). Nevertheless, the images at low concentration clearly indicate the presence of a network, which should lead to an elastic-like mechanical response.

**Figure 3.**
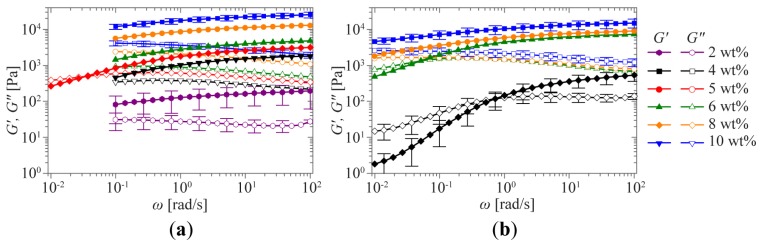
Oscillatory rheological measurements. Frequency sweeps for **1a** (**a**) and **1b** (**b**) at concentrations ranging from 2 and 4 to 10 wt %. Error bars shown on the curves for the highest and lowest concentrations indicate a typical experimental error, as quantified by the standard deviations of several independent measurements on the sample.

These expected changes in the mechanical properties were studied more closely by performing oscillatory rheological measurements. The concentration dependence of the mechanical properties of **1a** and **1b** was determined by performing frequency sweeps at concentrations ranging from 2 to 10 wt % in a frequency range between 0.01 and 100 rad s^−1^ and at a fixed 1% strain amplitude (Figure 3).

Hydrogelator **1a** exhibits a solid-like response at all concentrations measured. The storage modulus *G*′ is almost independent of the angular frequency *ω* and remains larger than the loss modulus *G*″ over the entire range of frequencies studied (Figure 3a). The high frequency plateau modulus *G*_∞_, taken as the storage modulus at the highest frequency in the scaled frequency plots, sensitively depends on concentration *c*. For the concentrations studied, a variation over 2 orders of magnitude is observed, ranging from 111 Pa at 2 wt % to 22 kPa at 10 wt %. However, because the network in the materials is bound by physical cross-links, a slow structural relaxation τ_r_, typified by a *G*′-*G*″ crossover (*G*′ = *G*″), is eventually expected to occur at long timescales.

**Figure 4.**
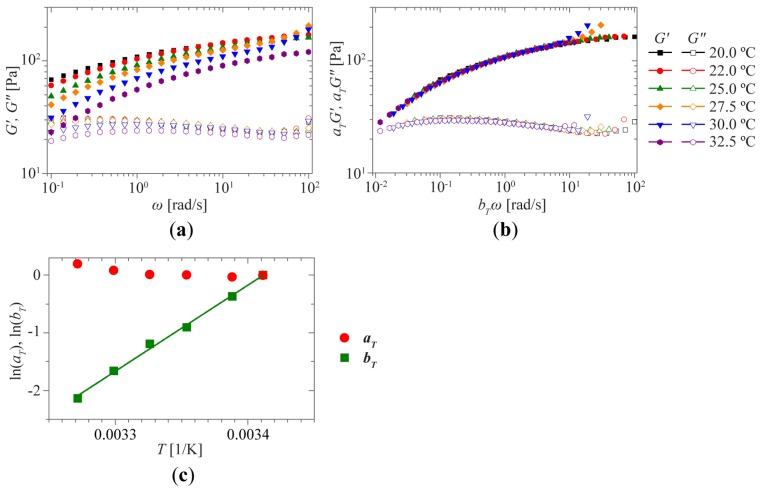
Time-Temperature Superposition for **1a** at a concentration of 2 wt %. Rescaling of the original curves for **1a** (**a**) at different temperatures ranging from 20 to 32.5 °C was performed using temperature-dependent shift factors *a*_T_ and *b*_T_ for the moduli *G*′ and *G*″ and the frequency *ω*, respectively. These shift factors are plotted (**c**) in an Arrhenius plot. The solid green line shows the linear fit to obtain the slope *E_a_*/*R*.

Typically, relaxation processes in polymers are sped up as the temperature is increased, without changing the shape of the distribution of relaxation times. As a result, the shape of the viscoelastic response of these materials remains unchanged as the temperature is increased. However, the response is shifted towards higher frequencies, reflecting the faster relaxation times. This so-called Time-Temperature Superposition (TTS) enables access to the viscoelastic response of the material over a dramatically increased range of frequencies. TTS is well-known for classical polymer systems, and generally works if the thermal motions of chains/molecules alone determine the relaxation in the material. However, in supramolecular systems the situation is more complex, as temperature does not merely affect the thermal motion of the polymer chains, but it actually modifies the molecular weight distribution of the polymer. As a result of this, application of TTS to supramolecular systems is not straightforward [29]. Nevertheless, we have attempted TTS on a 2 wt % sample of **1a** and studied the system up to the de-gelation temperature. However, no *G*′-*G*″ crossover could be observed, as shown in Figure 4a. Interestingly, the frequency shift factors *b*_T_ follow Arrhenius type behavior (Figure 4c), while the modulus shift factors *a*_T_ are found to be almost temperature independent. The derived activation energy *E*_a_ of 124 kJ mol^−1^ is similar to those observed for other transient networks in water [15,30,31], indicating that the slow relaxation can be attributed to the hydrophobic interactions between the UPy-groups.

**Figure 5.**
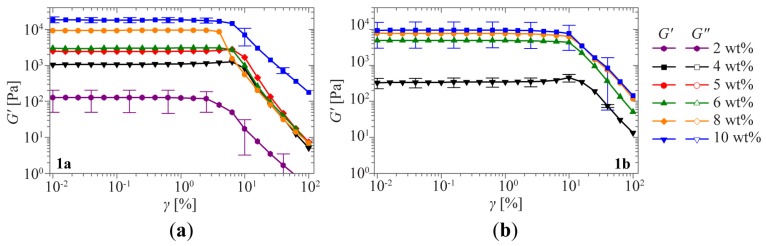
Oscillatory rheological measurements. Strain sweeps for **1a** (**a**) and **1b** (**b**) at concentrations ranging from 2 and 4 to 10 wt %. Error bars shown on the curves for the highest and lowest concentrations (at every third point) indicate a typical experimental error.

We attribute the immediate flow in the vial inversion test at low concentrations to the fact that the material has a low yield stress at low concentrations (see strain-dependent measurement, Figure 5a) and therefore yields under its own weight. However, in rheology no concentration-dependent sol-gel transition is observed. Indeed, a simple estimate of the gravitational shear stress in a vial with a diameter of 1 cm yields σ_vial_ ≈ ρ*gd/4* ≈ 25 Pa, with ρ the mass density of the sample and *g* the gravitational acceleration. For the lowest concentration samples this value exceeds the yield stresses as determined by tangent analysis (Figure 5a). This illustrates the shortcomings of the vial inversion method in the study of sol-gel transitions, as all these samples exhibit solid-like behavior when studied in linear oscillatory rheology. Detailed rheological measurements on these samples are thus essential.

**Figure 6.**
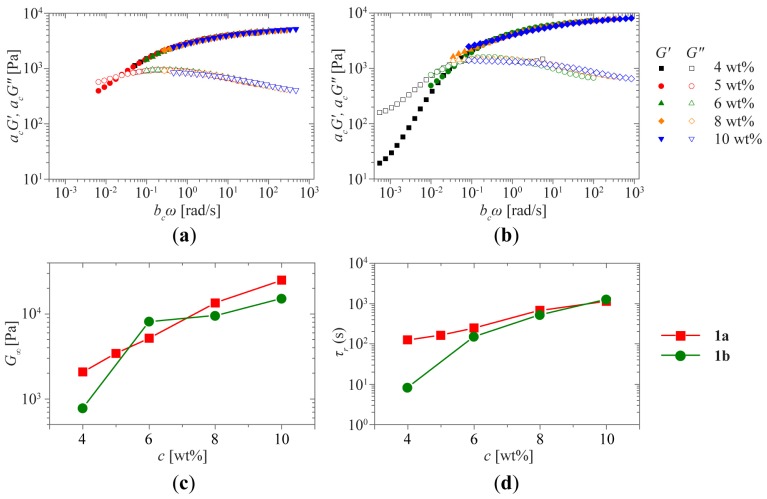
Time–Concentration Superposition. Rescaling of the original curves for **1a** (**a**) and **1b** (**b**) at concentrations ranging from 4 to 10 wt % was performed using concentration-dependent shift factors *a*_c_ and *b*_c_ for the moduli *G*′ and *G*″ and the frequency *ω*, respectively. These shift factors were used to determine the high frequency moduli *G*_∞_ (**c**) and relaxation time τ*_r_* (**d**) for **1a** (red squares) and **1b** (green circles) as function of concentration *c*, using the 6 wt % curve as reference condition for both compounds. Connecting lines are added to guide the eye.

The frequency sweeps of **1a**, measured at different concentrations, can be superposed onto a single master curve by rescaling the moduli and the strain amplitudes with concentration-dependent shift factors (Figure 6a). Our data thus exhibit Time-Concentration Superposition (TCSP), where the shape of the response is independent of concentration *c*. Thus, while an increase in concentration clearly leads to a slowing-down of relaxation processes in the material, the distribution and shape of these relaxations remains nearly unchanged. Similar behavior is observed at all concentrations, except for the 2 wt % sample, which could not be superposed onto the master curve. The modulus shift factors obtained from the master curve immediately reveal the dependence of the high frequency modulus *G*_∞_ and relaxation time τ*_r_* on concentration. As expected, *G*_∞_ increases with concentration *c* due to an increased density of the polymer network. The master curve could not be fitted with a single Maxwell model, corresponding to single exponential decay. Instead the data are more closely fit by a stretched exponential decay, showing that the material properties are not determined by a single relaxation time but governed by a broader range of relaxation times.

Hydrogelator **1b** also shows a viscoelastic solid-like response, but only at higher concentrations (Figure 3b). At lower concentrations, the relaxation time is on the order of 10 s, entering the experimental time frame of the vial inversion test and behaving like a viscoelastic fluid, as can be observed by the clear shifting of τ_r_ to higher frequencies when the concentration is lowered. Interestingly, the limiting low-frequency power law behavior for solutions (*G*′~*ω*^2^ and *G*″~*ω*^1^) is not observed, indicating the absence of simple fluid-like behavior at lower concentrations. At the lowest frequencies measured for 2 wt % **1b**, an upturn in both moduli can be observed, which can be attributed to drying of the sample during the measurement time required for measuring at low frequencies. The superposition of the curves is not as evident as for **1a**, suggesting that the nature of the viscoelastic behavior at low concentrations might change due to a change in the network structure. Similar strain-dependent behavior is observed for **1b** (Figure 5b).

The main characteristics of both hydrogelators can be summarized by investigating the concentration-dependence of their plateau moduli *G*_∞_ (the modulus at high frequency, Figure 6c) and their characteristic relaxation times τ_r_ (defined by the crossover of *G*′ and *G*″, Figure 6d). Comparing the two materials it becomes evident that at concentrations above 5 wt % both materials exhibit very similar behavior. In general, **1b** exhibits a lower *G*_∞_ than **1a**, which we hypothesize to be due to the fact that, at the same concentration, **1b** has less UPy-groups available for cross-linking than **1a** has. However, at lower concentrations, larger deviations occur, which we hypothesize to be due to a similar disparity in the number of available cross-linking groups. This indicates that the **1b** system is closer to the critical concentration where a percolating network structure can be formed in the material.

Analysis of both the magnitude of *G*_∞_ and the *G*′-*G*″ crossover as a function of frequency are used to characterize the scaling behavior of the sytem. The plateau moduli exhibit power-law scaling, *G*_∞_ ∝ *c^n^*, with exponents *n* of 2.7 and 3.0 for **1a** and **1b**, respectively. These values correspond within experimental error to the exponent of 5/2 found for the theory of densely crosslinked gels of semiflexible polymers [32] and experimentally found for neurofilaments [33] and actin [34]. In this short-time regime, we would indeed not expect the nature of the cross-links to affect the behavior. Here transient networks should exhibit the same behavior as permanently cross-linked systems. Models for permanently cross-linked or even entangled semi-flexible chains are thus appropriate to account for this high frequency behavior. We however do not expect this theory to apply to our system at the much lower frequencies corresponding to the typical relaxation times of the material. The relaxation time τ_r_ scales with concentration as a power-law with exponent 2.5 and 5.5 for **1a** and **1b**, respectively. Unfortunately, to our knowledge, there is no theory available that predicts the behavior of transient, semiflexible networks at low frequencies. Theories have been developed that predict scaling laws of the terminal relaxation for transient, flexible polymers [35–37], where an exponent of 2.44 is predicted for entangled strands at high concentration. However, to our knowledge, these theories have not been adapted to semiflexible polymers, and cannot account for the exponent of 5.5 found for **1b**.

**Figure 7.**
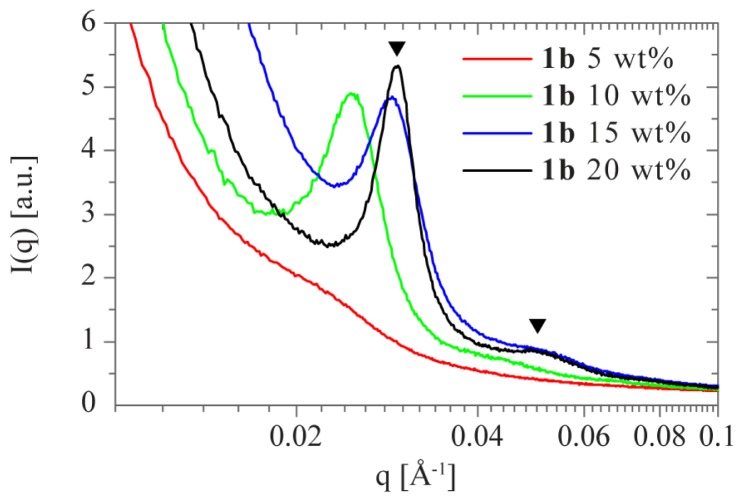
Small-angle X-ray scattering profiles of **1b** at 5 (red), 10 (green), 15 (blue) and 20 wt % (black) samples measured in PBS at room temperature. Solid triangles correspond to the allowed reflections (*q* = q*(1, √3)) for hexagonal structures.

Small-angle X-ray scattering (SAXS) experiments were performed on **1b** to study the structure of the hydrogels in the concentration range 5–20 wt % (Figure 7). Swollen gels are often intrinsically heterogeneous, and show two correlation lengths, ξ_m_ and ξ_h_ [38]. ξ_m_ depicts the mesh size of the polymer network (typically a few nm), while ξ_h_ is a measure for the structural heterogeneities on larger length scales (often tens of nm). The steep slope at low *q*-values in all SAXS profiles in Figure 7 indicates that ξ_h_ is outside the accessible *q*-range, indicating the presence of microscale domains which are larger than 2π/*q*_min_ ~ 59 nm. Interestingly, the SAXS profiles of 10–20 wt % **1b** exhibit an additional feature in the intermediate *q*-range, which we attribute to the average distance between the supramolecular aggregates. Further contributions can be expected from the form factor and the structure factor of the ordering of the supramolecular aggregates. The SAXS profile is dominated by a broad peak due to the structure factor, which prevents extracting ξ_m_ from our measurement.

The corresponding intermicellar distances, *d*, follow from Bragg’s law, *i.e.*, *d* = 2π/*q*_max_, and the position of the broad correlation peak at *q*_max_. We found *d* = 25.4, 21.8 and 21.4 nm for 10, 15 and 20 wt % **1b**, respectively. As expected, *d* decreases with increasing concentration as the polymer volume fraction in the gel increases. The interaction peak is least pronounced and most broad at 10 wt %. A second weak and broad reflection appears at *q* ~ √3*q*_max_ for 15 and 20 wt % **1b** suggesting a hexagonal packing upon increasing concentration, as expected for fiber-like structures. In summary, the correlation peaks at 0.0247 < *q*_max_ < 0.0294 Å^−1^ arise from the interaction between cylindrical polymer micelles and may be attributed to the average spacing between the hydrophobic domains, which decreases and becomes more defined upon an increase in polymer volume fraction. The minimal separation is related to the size of the PEG spacer used (*R*_g_ ~ 6.2 nm for **1b**) [9,24]. Similar trends have been reported previously by others for photopolymerized PEG networks and triblock copolymer gels [22,39,40].

### 2.2. Sol-Gel Transition Triggered by Temperature

**Figure 8.**
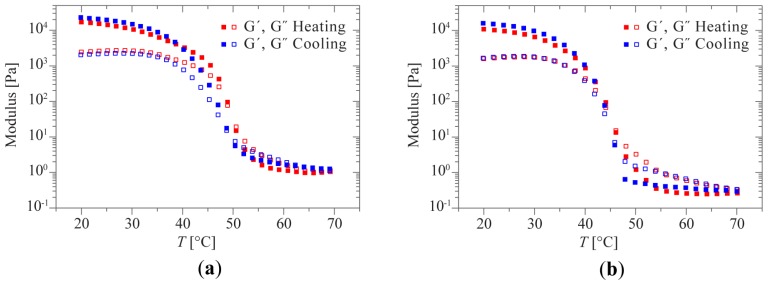
Temperature dependence of *G*′ and *G*″ for 10 wt % samples of **1a** (**a**) and **1b** (**b**), measured at 10% strain amplitude, an angular frequency of 1 Hz and a heating and cooling rate of 1 °C/min.

**Figure 9.**
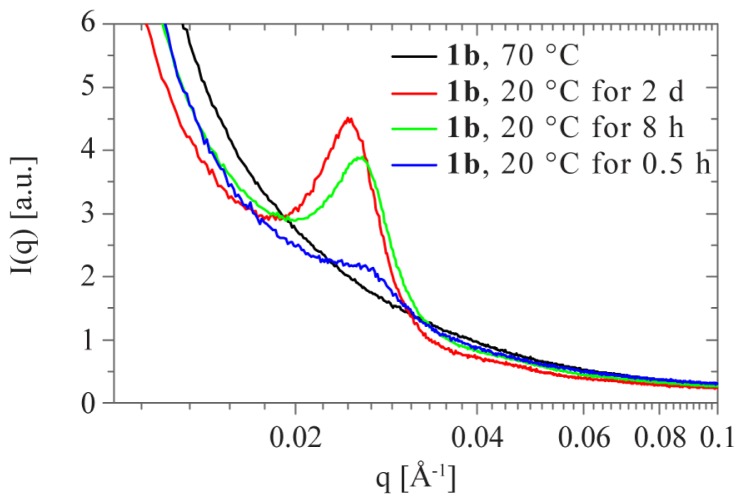
Small-angle X-ray scattering profiles of **1b** at 10 wt % at 70 °C (black) and after 2 days (red), 8 h (green) and 0.5 h (blue) after cooling back to room temperature.

In previous studies, drug-loaded gels were successfully prepared by addition of drugs into polymer solutions at elevated temperatures and subsequent cooling to room temperature [21]. This implies that an increase in temperature triggers a sol-gel transition. Indeed, sharp transitions from high to low moduli are observed in temperature-dependent oscillatory rheology. The crossover of *G*′ and *G*″ evidences a sol-gel transition occurring around 50 and 45 °C for **1a** and **1b**, respectively (Figure 8). This transition is fully reversible and occurs within the time-frame of the measurement (*i.e.*, within ~1 min). Fully in line with the behavior observed in rheology, the structure peak that is visible in the SAXS profiles of 10 wt % **1b** at room temperature vanishes at 70 °C and reappears upon cooling (Figures 9 and S2). This clearly reflects the relation between the mesoscopic ordering within the gel and its mechanical behavior. The interaction peak reappears within 30 minutes after cooling, yet the intensity increases during several hours. Full recovery of mesoscopic order is slower in SAXS compared to the recovery of the mechanical properties observed in our rheological measurements. It thus appears that in this regime the degree of ordering no longer significantly affects the linear viscoelastic properties.

### 2.3. Sol-Gel Transition Triggered by pH

**Figure 10.**
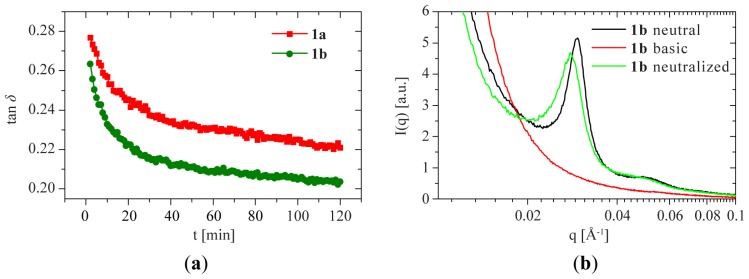
pH dependence of the mechanical properties and of the SAXS profile. (**a**) Gel curing followed by plotting tan δ, as a function of time for **1a** (green squares) and **1b** (red circles) at 10 wt %; (**b**) Small-angle X-ray scattering profiles of **1b** at 10 wt % under neutral (black), basic (red) and neutralized (green) conditions.

As reported earlier, gels of **1b** can be reversibly switched from a fluid to a gel state using pH [18]. Under basic conditions (0.1 M NaOH), the samples behave like a fluid and no storage modulus can be measured in rheology. Instantaneous gelation is observed after neutralizing the sample (Figure 10a). The formation of the gel is followed by plotting tan δ = *G*″/*G*′ as a function of time. As evidenced by tan δ < 1, immediately after addition of acid, the material shows the characteristics of a solid-like network. It takes on the order of a few hours for the gel to obtain its final strength, which is similar to the gels prepared without switching the pH. As anticipated, the structure peak that is present in the SAXS profiles of 10 wt % **1b** under neutral conditions disappears for 10–20 wt % **1b** under basic conditions and returns to its original intensity and position once the samples are neutralized (Figures 10b and S3). Fully in line with the rheology, this demonstrates the reversibility of the sol-gel transition.

Previously, we proposed that the transition from gel to sol upon an increase in pH is due to the deprotonation of the enol tautomer of the UPy-group [18], rendering the hydrophobic block more water-soluble, leading to the “dissolution” of the physical cross-links between the self-assembled fibers such that the hydrogel transforms into a liquid-like material. This process is visible both in rheology and in SAXS as the mechanical response becomes liquid-like and the structural feature related to the spacing between hydrophobic domains disappears. Upon addition of acid, the enol tautomer returns to the keto tautomer enabling the UPy-groups to dimerize, which restores the structural order and the physical cross-links, as evidenced by the reappearance of the interaction peak in SAXS and the formation of a solid-like network in rheology, respectively.

## 3. Experimental Section

### 3.1. Materials

All solvents were obtained from Sigma-Aldrich and used as received. Water was deionized prior to use. The polymers were synthesized by SyMO-Chem BV, Eindhoven, The Netherlands.

### 3.2. Methods

#### 3.2.1. Rheology

Mechanical properties were assessed on a stress-controlled rheometer (Anton Paar GmbH, Physica MCR501, Ostfildern, Germany) equipped with a parallel-plate geometry (PP-25). All measurements were performed at 20 °C unless specified otherwise. A solvent trap was used to prevent evaporation of the solvents. First, the linear viscoelastic regime was determined using a strain sweep experiment at an oscillation frequency of 1 rad s^−1^. Frequency sweeps were performed at 1% strain amplitude. Gel curing was measured at 0.5% strain amplitude at a frequency of 1 rad s^−1^. Samples were loaded as solutions in 0.1 M NaOH and neutralized with 1 M HCl in the rheometer and left to equilibrate for 2 h before starting the measurement, except when measuring the buildup, in which case the measurement was started immediately.

#### 3.2.2. Small-Angle X-ray Scattering

The small-angle X-ray scattering (SAXS) measurements were performed at the cSAXS–X12SA beamline at the Paul Scherrer Institute in Villigen, Switzerland, operating at 12.4 keV. The sample to detector distance was 8 m. Two-dimensional scattering patterns were recorded on a 2D Pilatus 2M detector with a pixel size of 172 × 172 μm^2^. The 2D images were normalized to the intensity of the incident beam and radially averaged to obtain 1D SAXS profiles (*I*(*q*) *vs. q*) where the scattering vector *q* is given by *q* = 4π(sinθ/2)/λ with the radiation wavelength, λ, and the scattering angle, θ. The 1D SAXS profiles were corrected for background scattering from the quartz capillary and for electronic noise. Samples were prepared by dissolution of the polymer in basic medium (water, PBS or 0.1 M NaOH) and neutralized with 1 M HCl. Samples were loaded in 1 mm quartz capillaries (Hilgenberg GmbH, Malsfeld, Germany). Room temperature gels were loaded at 70 °C and left to equilibrate at room temperature for 30 min up to 2 days before the measurements. Heating was performed by connecting the sample holder to a heat bath at 70 °C and cooling was performed by air. Samples were heated again to 70 °C before every time step.

#### 3.2.3. Confocal Microscopy

Confocal microscopy was performed on a Leica SP5 inverted confocal microscope (Leica Microsystems, Rijswijk, The Netherlands) with a 40× water immersion objective. Samples were prepared by dissolution of the polymer **1a** in 0.1 M NaOH solution. The solution was transferred to wells and 1 M HCl solution was added to neutralize the sample. The final concentration of the polymer was 1 wt %. Nile Red (purchased from Sigma Aldrich, Zwijndrecht, The Netherlands) at a final concentration of 5 μM was used as lipophilic dye to visualize the hydrophobic domains in the polymer network. Images were obtained by illumination of the sample with a laser at 550 nm and collecting fluorescence from 575 to 700 nm.

## 4. Conclusions

Herein we report on multi-responsive supramolecular hydrogels composed of poly(ethylene glycol), end-functionalized with ureido-pyrimidinone fourfold hydrogen units. Rheology and SAXS experiments were performed to obtain more insight into the effects of environmental triggers, such as concentration, temperature and pH, on the structure and mechanics of these supramolecular hydrogels. Oscillatory rheology reveals that each of these triggers can be used to generate a sol-gel transition as evidenced by, e.g., a sharp reduction and cross-over of the loss and storage moduli. Interestingly, this mechanical response is coincident with the (dis)appearance of an interaction peak in the SAXS profiles, which we attribute to the average spacing between physically cross-linked hydrophobic domains.

Upon increasing the concentration, we observe an increasingly ordered structure in the samples in SAXS. The correlation peak arising from the interaction between polymer micelles becomes more defined as the average spacing decreases and becomes more uniform. The corresponding mechanical transition from a liquid-like solution to a gel is gradual, and a clear-cut onset of elastic-like behavior is absent. Instead, our experiments indicate a slow structural relaxation, due to hydrophobic interactions, is always present with a characteristic relaxation time that scales with concentration as a weak power-law representing densely crosslinked semiflexible polymers. The onset of elastic-like behavior thus depends on the time scale at which the response is studied, and as a result only gradual changes in viscoelastic behavior are observed when the concentration changes.

**Figure 11.**
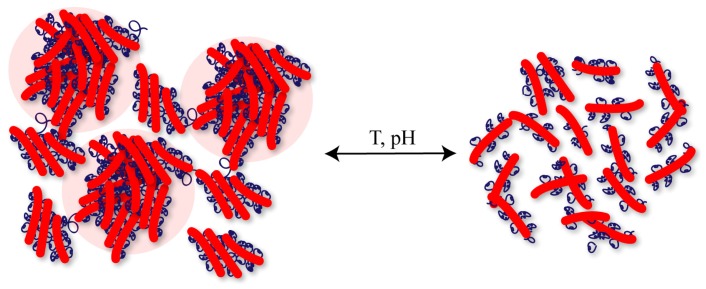
Schematic representation of the gel with microscopic domains of densely cross-linked polymers. Addition of base, or increase in temperature, renders the individual polymer stacks more water-soluble, and induces the disappearance of the cross-links and the mesoscale domains.

However, a sharp transition was observed in rheology and the structural feature in SAXS disappeared when the temperature was increased. Increasing the pH also causes the structural feature in SAXS to disappear and a cross-over of the storage and loss moduli in rheology. We hypothesize that both triggers enhance the water-solubility of the hydrophobic block, leading to “dissolution” of the physical cross-links between polymer micelles, which renders the average domain spacing less defined (Figure 11).

These results provide more insights into the mechanism of the network formation in these materials, which can be exploited for tailoring the behavior of these systems for biomedical applications, where any of the triggers discussed might be used. These additional valuable insights into the structure-property relationship in supramolecular hydrogels can aid the development of tough, strong hydrogels for application in regenerative medicine.

## Supplementary Information
