# Supplementary material for: Mesoscale Characterization of Supramolecular Transient Networks Using SAXS and Rheology
Source: Int J Mol Sci. 2014 Jan 16;15(1):1096–111. doi: 10.3390/ijms15011096 (PMC3907858; doi:10.3390/ijms15011096)
Supplement: Supplementary file 1 [file ijms-15-01096-s001.pdf]

Supplementary Information

Figure S1. Confocal microscopy image of a viscoelastic solution of **1a** at a concentration of 5 wt% under neutral conditions. The scale bar corresponds to 50 µm.

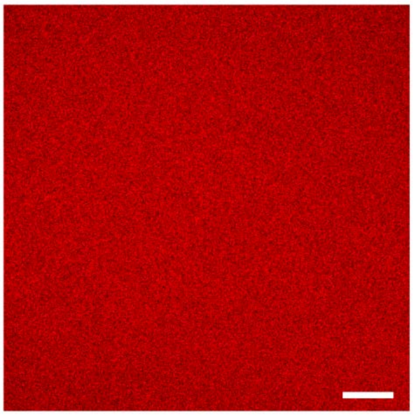

Figure S2. Small-angle X-ray scattering profiles of **1b** at (**a**) 5 wt %, (**b**) 15 wt % and (**c**) 20 wt % at 70 °C (black) and 2 days (red), 8 h (green) and 30 min (blue) after cooling back to room temperature.

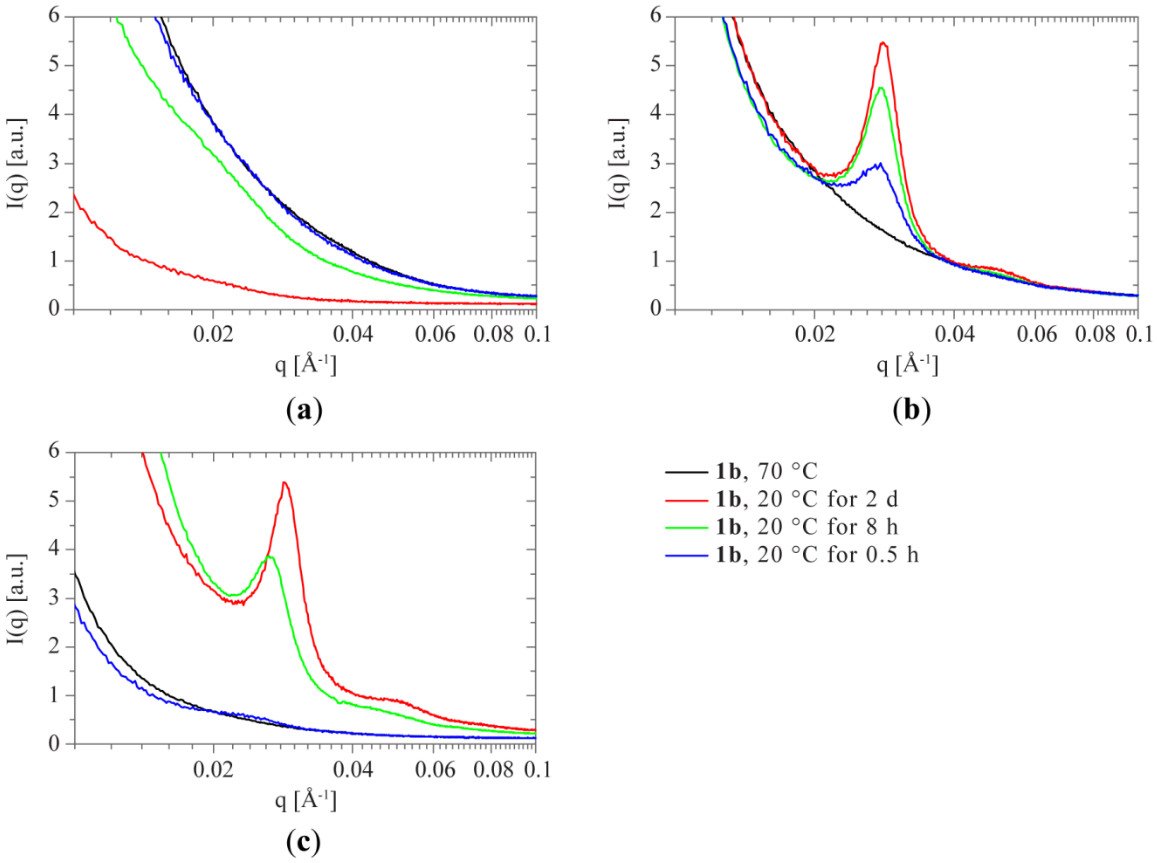

Figure S3. Small angle X-ray scattering profiles for **1b** at (**a**) 5 wt %, (**b**) 15 wt % and (**c**) 20 wt % under neutral (black), basic (red) and neutralized (green) conditions.

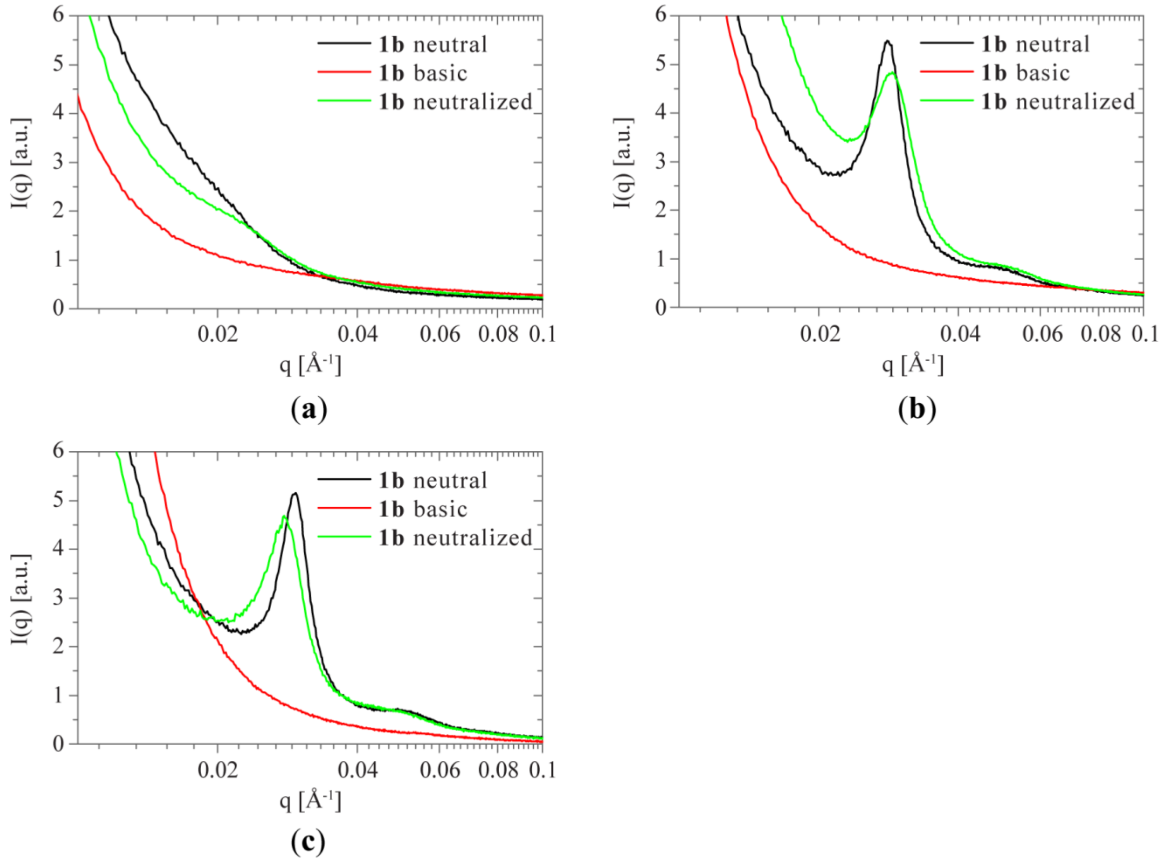

© 2014 by the authors; licensee MDPI, Basel, Switzerland. This article is an open access article distributed under the terms and conditions of the Creative Commons Attribution license (http://creativecommons.org/licenses/by/3.0/).
